# Sodium nitroprusside improved the quality of Radix Saposhnikoviae through constructed physiological response under ecological stress

**DOI:** 10.1038/s41598-023-43153-3

**Published:** 2023-09-22

**Authors:** Xiao-Wen Song, Yao Yao, Peng-Cheng Yu, Wei Zhang, Wen-Fei Liu, Li-Yang Wang, Kai Zhao, Jin-Cai Lu, Xiang-Cai Meng

**Affiliations:** 1 College of Pharmacy, Heilongjiang University of Chinese Medicine, Harbin, 150040 China; 2 Shenyang Pharmaceutical University, Shenyang, 110016 China

**Keywords:** Chemical biology, Plant sciences

## Abstract

The ecological significance of secondary metabolites is to improve the adaptive ability of plants. Secondary metabolites, usually medicinal ingredients, are triggered by unsuitable environment, thus the quality of medicinal materials under adversity being better. The quality of the cultivated was heavily declined due to its good conditions. Radix Saposhnikoviae, the dried root of *Saposhnikovia divaricata* (Turcz.) Schischk., is one of the most common botanicals in Asian countries, now basically comes from cultivation, resulting in the market price being only 1/10 to 1/3 of its wild counterpart, so improving the quality of cultivated Radix Saposhnikoviae is of urgency. Nitric oxide (NO) plays a crucial role in generating reactive oxygen species and modifying the secondary metabolism of plants. This study aims to enhance the quality of cultivated Radix Saposhnikoviae by supplementing exogenous NO. To achieve this, sodium nitroprusside (SNP) was utilized as an NO provider and applied to fresh roots of *S. divaricata* at concentrations of 0.03, 0.1, 0.5, and 1.0 mmol/L. This study measured parameters including the activities of antioxidant enzymes, secondary metabolite synthesis enzymes such as phenylalanine ammonia-lyase (PAL), 1-aminocyclopropane-1-carboxylic acid (ACC), and chalcone synthase (CHS), as well as the contents of NO, superoxide radicals (O_2_^**·**−^), hydrogen peroxide (H_2_O_2_), malondialdehyde (MDA), and four secondary metabolites. The quality of Radix Saposhnikoviae was evaluated with antipyretic, analgesic, anti-inflammatory effects, and inflammatory factors. As a result, the NO contents in the fresh roots were significantly increased under SNP, which led to a significant increase of O_2_^**·−**^, H_2_O_2_, and MDA. The activities of important antioxidant enzymes, including superoxide dismutase (SOD), catalase (CAT), and peroxidase (POD), were found to increase as well, with their peak levels observed on the 2nd and 3rd days. PAL, ACC, and CHS activities were also significantly enhanced, resulting in the increased secondary metabolite contents of Radix saposhnikoviae in all groups, especially the 0.5 mmol/L SNP. The four active ingredients, prim-*O*-glucosylcimifugin, cimifugin, 4′-*O*-β-d-glucosyl-5-*O*-methylvisamminol, and sec-*O*-glucosylhamaudol, increased by 88.3%,325.0%, 55.4%, and 283.8%, respectively, on the 3rd day. The pharmaceutical effects of Radix Saposhnikoviae under 0.5 mmol/L SNP were significantly enhanced. Exogenous SNP can induce the physiological response of *S. divaricata* under adverse conditions and significantly improve the quality of Radix Saposhnikoviae.

## Introduction

Radix Sposhnikoviae is a widely used herbal medicine in Asian countries, derived from the root and rhizome of the plant S*aposhnikovia divaricata* (Trucz.) Schischk, belonging to the Umbelliferae family^1^. This medicinal plant is rich in various bioactive components, including chromone, coumarin, and polysaccharides. The pharmacological activities of Radix Sposhnikoviae are diverse and valuable, such as antipyretic, analgesic, anti-inflammatory, rheumatic, anti-tumor, and antioxidant^2^. With the increase in population and farmland, as well as years of over-harvesting, the wild resources of *S. divaricata* have been depleted and cannot meet the market requirements of more than 5000t per year hence the cultivated have become a primary source, which accounts for more than 70% of the market share^3^. The transformation from wild to cultivated has also led to a serious decline in quality, with the active ingredient content reduced by half^3,4^ and the price being only 1/3 to 1/10 of that of wild, making the improvement of the cultivated Radix Saposhnikoviae quality to an urgent issue^5^. The quality of herbs directly influences their healing effects. Today, researchers emphasize evaluating various plant constituents to understand their pharmacological effects and explore their potential healing properties^6^.

It is worth noting that unfavorable environmental conditions can contribute to variations in the quality of herbal medicine^7^, while moderate ecological stress is an indispensable condition for producing high-quality herbal medicine. Photosynthesis in plants is the process of biosynthesis, CO_2_ and H_2_O into energy-rich organic matter through the absorption of light energy, electron transfer, photosynthetic phosphorylation, and carbon assimilation while releasing O_2_. Reactive oxygen species (ROS), including O_2_^**·**−^, H_2_O_2_, **·**OH, etc., are hyperactive oxygen-containing compounds produced during this aerobic metabolism in organisms: the released O_2_ is easily reduced to the chemically hyperactive O_2_^**·**−^, then transformed into **·**OH, H_2_O_2_, etc. It has been confirmed that under severe ecological stress, plants generally experience a significant increase in ROS levels^8^, resulting in various physiological and metabolic alterations^9^, further leading to damage in essential macromolecules, such as proteins, DNA and biofilm^10^. During the protracted revolution, organisms evolved antioxidant enzyme systems such as superoxide dismutase (SOD), catalase (CAT), and peroxidase (POD) et al. to eliminate excess ROS and lessen their damage. Nevertheless, unlike mobile organisms, plants cannot avoid adversity by moving, and large amounts of ROS will inevitably be produced, making it difficult for plants to adapt to adversity solely through antioxidant enzymes. To enhance their adaptability to the environment, plants have evolved secondary metabolism as a supplement. Chemical bonds such as –S–S-bonds are indispensable for enzymes to maintain the corresponding structure and carry out catalysis. Because excess ROS can affect the formation of these chemical bonds, it can affect metabolism by the altered the activity of enzymes, and become the medium of the environment influencing metabolism. It has been reported that ROS can promote the change of plant antioxidant enzymes and secondary metabolites at a certain concentration^9^. Secondary metabolites are significant in plant adaptation to ecological stress, and these metabolites often serve as the active ingredients of herbal medicine^11^. Numerous studies have demonstrated that the high amount of ROS under ecological stress can elevate the contents of plant secondary metabolites^12^, for example, the active ingredient contents of *Scutellaria baicalensis* increased by more than 50% when exposed to Na_2_S_2_O_4_ (a carrier substance for O_2_^**·**−^). Similarly, the quality of Fructus schisandrae chinensis was significantly improved when treated with H_2_O_2_, etc.^13,14^.

NO is a small molecule free radical with a high solubility in water and lipids, allowing it to diffuse across biological membranes into adjacent tissue cells freely. Through processes such as oxidation–reduction of electron carrier, ATP synthase, changes in mitochondrial membrane potential, and complex reverse electron transport, NO can produce O_2_^**·**−^^15^, making it capable of exhibiting the effects of ROS. Additionally, NO has four main physiological effects: (1) Reducing oxidative damage: NO can rapidly convert the higher activity O_2_^**·**−^ to low activity ONOO^**·**^ for plants^15–18^, with a reaction rate of 5 × 10^9^ mol L^−1^ s^−1^. Meanwhile, it can significantly reduce the conversion of O_2_^**·**−^ to ·OH, the destruction of the latter being about thousands of times more than O_2_^**·**−^. (2) Metabolic regulation: NO can regulate enzyme activities through protein modification by binding to cysteine residues, hemoglobin, or iron-sulfur centers, as well as through ONOO˙ formation and tyrosine residue nitration^17^. (3) Regulation of cellular redox environment: NO maintains metabolic homeostasis by decreasing GSH/GSSG^19,20^. (4) Eliminate ROS. NO is highly active and quickly reacts with O_2_, H_2_O_2_, etc., making it less destructive. As a result, NO has a broader advantage. NO can improve the antioxidant capacity of chickpea plants, the antioxidant and wound-healing ability of sweet potato roots, and more^21,22^. Additionally, NO and ROS can synergistically improve plant resistance and alleviate the damage caused by ecological stress^23^. Sodium nitroprusside (SNP), as an exogenous NO donor, is capable of generating both NO and ROS in organisms^24^ and leads to oxidative stress. Still, it also contributes to the imbalance of ROS and potential damage to cells and tissues^25^. On the positive side, SNP can also improve the tolerance of plants to ecological stress, with total phenols and flavonoids being significantly elevated in tomatoes^26^. Additionally, for marjoram herb, SNP treatment substantially enhances secondary metabolite content and essential oil yield^27^. Notably, plants' fresh medicinal parts possess a complete secondary metabolism, and SNP can also directly influence isolated roots. In this study, the application of SNP elucidated the formation of Radix Saposhnikoviae quality by inducing a physiological response under ecological stress. This approach opens up new possibilities for producing high-quality Radix Saposhnikoviae and represents a promising avenue for further research and development in herbal medicine.

## Materials and instruments

### Samples

All the plant experiments complied with relevant institutional, national, and international guidelines and legislation. Cultivated Radix Saposhnikoviae (FF20221001) collection was done with permission. Fresh 3-year-old roots of cultivated *S. divaricata*, identified by Prof. Xiang-Cai Meng of Heilongjiang University of Chinese Medicine, were collected in Daqing City, Heilongjiang Province, China, collected in October 2022 and immediately wrapped in plastic to keep fresh. The cultivated Radix Saposhnikoviae (FF20221001) has been deposited in a publicly available herbarium of Heilongjiang University of Chinese Medicine. And we complied with the IUCN Policy Statement on Research Involving Species at Risk of Extinction and the Convention on the Trade in Endangered Species of Wild Fauna and Flora.

### Instruments

LC-2010A High-performance liquid chromatography (Japan), 752 UV–visible spectrophotometers (Shanghai Jinghua Scientific Instrument Co., Ltd.), CP225D 0.1 mg electronic balance (Shanghai Precision Instrument Co., Ltd.), SD40 ice machine (Guangzhou Guangkun Electrical Manufacturing Co., Ltd.), TGL-16LM desktop high-speed refrigerated centrifuge (Hunan Xingke Scientific Instrument Co., Ltd.), DKZ-3B water bath thermostat oscillator (Changzhou Xunsheng Instrument Co., Ltd.), Julabo TW20 digital display thermostatic water bath (Julabo Germany Co., Ltd.), QL-902 vortex oscillator (Haimen Qilin Bell Instrument Manufacturing Co., Ltd.), Thermo enzyme standard instrument (Thermo Limited, USA), DHG-9015A blast dryer (Shanghai-Hengke Scientific Instrument Co., Ltd.) Shimadzu.

### Reagent

O_2_^**·−**^ Free Radical Determination Kit (Beijing Solabo Technology Co., Ltd., 20221109); Protein Quantification (TP) Determination Kit (20220220), H_2_O_2_ Determination Kit (20220903), Superoxide Dismutase (SOD) Determination Kit (20220624), Catalase (CAT) Determination Kit (20220903), Peroxidase (POD) Determination Kit (20220616), Malondialdehyde (MDA) Determination Kit (20220422), Phenylalanine Ammonia-Lyase (PAL) Determination Kit (20220806) (Nanjing Jiancheng Bioengineering Research Institute); 1,3-Phosphoglyceric Acid (202211), NO Determination Kit (202211), ACC Determination Kit (202303), CHS Determination Kit (202303) (Jiangsu Jingmei Biotech Co., Ltd.); Prim-O-glucosylcimifugin (AF21092803), Cimifugin (AF21092804), 4′-*O*-β-d-glucosyl-5-*O*-methylvisamminol (AFBG 1328), Sec-*O* -glucosylhamaudol (AF20081401) were purchased from Chengdu Aofei Biotechnology Co., Ltd., with a purity greater than 98%; HPLC-grade methanol (R142190); Sodium nitroprusside (Zhengzhou Pani Chemical Reagent Plant); Rats and mice was purchased from Liaoning Changsheng Biotechnology Co., Ltd. by the Drug Safety Evaluation Center of Heilongjiang University of Chinese Medicine in Liaoning Changsheng Biotechnology; Dry yeast (Anqi Co., Ltd.), Glacial acetic acid (Tianjin Tiantli Chemical Reagent Co., Ltd.), xylene (Tianjin Fuyu Fine Chemical Co., Ltd.), and physiological saline (Harbin Sanlian Pharmaceutical Co., Ltd.) were also used; Mouse Tumor Necrosis Factor-α (TNF-α) ELISA Kit (202303), Mouse Interleukin-6 (IL-6) ELISA Kit (202303) (Jiangsu Jingmei Biotech Co., Ltd.), etc.

## Experimental method

### Sample handling

The collected fresh roots of *S. divaricata* were mixed evenly and then divided into 5 groups. SNP aqueous solution with 0.00, 0.03, 0.10, 0.50, and 1.0 mmol/L was sprayed, respectively, until the droplet was about to drop, that is, saturation, to ensure that the dosage could be controlled and uniform. The pre-test results showed that several active ingredients peaked on the 3rd day, suggesting that the desired effect of substance SNP was anticipated on the 2nd day. To match this expectation, we applied SNP only on the first two days and switched to distilled water afterward. The 0.30 g × 30 samples of fresh root phloem were taken from each group per day, and three repeat samples for each index were used to determine activities of SOD, POD, CAT, PAL, CHS, ACC, and the contents of malondialdehyde (MDA), 1,3-diphosphoglyceric acid (1,3-DPG), and NO. Fresh root phloem of each group was taken 0.10 g × 10 samples to determine O_2_^**·**−^ and H_2_O_2_. The above samples were sealed in aluminum foil and stored in a refrigerator at – 80 °C. At the same time, at least six fresh roots were taken from each group, homogeneous per sample, more than 30 g × 3, dried in an oven at 55 °C, then crushed and sieved through 60-mesh sieve for the determination of the contents of prim-*O*-glucosylcimifugin, cimifugin, 4′-*O*-β-d-glucosyl-5-*O*-methylvisamminol, and sec-*O*-glucosylhamaudol.

### Determination of NO contents

The NO contents in fresh roots were determined with a plant NO ELISA assay kit.

### Determination of ROS contents

The homogenized protein contents. The fresh roots were determined with the protein quantification (TP) assay kit Bradford method and the O_2_^**·**−^ and H_2_O_2_ using the O_2_^**·**−^ assay kit and the plant H_2_O_2_ assay kit, respectively.

### Determination of MAD contents

The MDA contents were determined with TBA using a malondialdehyde kit.

### Determination of antioxidant enzyme activities

The activities of antioxidant enzymes were determined with SOD, CAT, and POD assay kits, respectively.

### Determination of metabolites and key enzyme activities

The contents of primary metabolite 1,3-DPG were determined with a plant 1,3-DPG ELISA detection kit, the activities of PAL ACC and CHS using a PAL, Acetyl coenzyme A carboxylase (ACC), and chalcone synthase (CHS) ELISA kits, respectively.

### Determination of main active ingredient contents

#### Solution preparation

Preparation of standard solution: Accurately weigh 1.88 mg of prim-*O*-glucosylcimifugin and 2.25 mg of 4′-*O*-β-d-glucosyl-5-*O*-methylvisamminol into a 5 mL volumetric flask, and methanol was added to the scale to prepare 0.38 mg/mL and 0.45 mg/mL of the control solution. In addition, accurately weigh 1.03 mg of cimifugin and 1.05 mg of sec-*O*-glucosylhamaudol into a 25 mL volumetric flask, and methanol was added to the scale to make up 0.042 mg/mL, and 0.021 mg/mL of the control solution. Preparation of sample solution: Accurately weigh 1.5 g of the three groups of Radix Saposhnikoviae into a 50 mL conical flask, 50 mL of methanol was added, and the mass was weighed and extracted by heating reflux at 70 °C for 2 h in a water bath. After cooling, weigh it again, balance the weight loss, shake well, and filter. The filtrate was then passed through a microporous membrane (0.45 μm), and the contents of prim-*O*-glucosylcimifugin, cimifugin, 4′-*O*-β-d-glucosyl-5-*O*-methylvisamminol, and sec-*O*-glucosylhamaudol were determined with HPLC.

#### Chromatographic conditions

Diamonsil C_18_ chromatographic column (250 mm × 4.6 mm, 5 μm); column temperature: 40 °C; mobile phase: methanol (A)-aqueous solution (B); elution gradient: 0–8 min, 30–50% A; 8–18 min, 50–70% A; volume flow rate: 1.0 mL/min; detection wavelength: 254 nm, injection volume: 10 μL.

#### Methodological investigation

The intra-day precision was calculated by taking the test solution under 2.7.1 and measuring it 6 times according to the method under 2.7.2; the inter-day precision was calculated by analyzing it for three consecutive days. The RSDs of the inter-day precision of prim-*O*-glucosylcimifugin, cimifugin, 4′-*O*-β-d-glucosyl-5-*O*-methylvisamminol, and sec-*O*-glucosylhamaudol were 1.35%, 1.63%, 1.25%, and 1.67%, respectively, indicating the precision was high.

The test solution under 2.7.1 was taken and determined with the method under item 2.7.2 to obtain the RSDs of the test solutions were 0.28%, 2.75%, 1.07%, and 2.79% for prim-*O*-glucosyl cimifugin, cimifugin, 4′-*O*-β-d-glucosyl-5-*O*-methylvisamminol, and sec-*O*-glucosyl hamaudol, respectively, indicating good repeatability of the analytical method.

The RSDs of peak areas of prim-*O*-glucosylcimifugin, cimifugin, 4′-*O*-β-d-glucosyl-5-*O*-methyl visamminol, and sec-*O*-glucosylhamaudol were 1.76%, 1.78%, 0.79%, and 1.64%, respectively, which indicated that the stability of the test solution was good within 12 h. The test solution was analyzed under 2.7.2 for 0 h, 3 h, 6 h, 9 h, and 12 h after preparation.

The average recoveries were 98.60%, 101.08%, 100.23%, and 99.90% with RSDs of 2.69%, 2.44%, 2.02%, and 1.80%, respectively, when the known content of Radix Saposhnikoviae from Daqing was added with a prim-*O*-glucosylcimifugin, cimifugin, 4′-*O*-β-d-glucosyl-5-*O*-methyl visamminol, and sec-*O*-glucosylhamaudol controls.

### Pharmacological effects verification

#### Drug preparation

The medicinal efficacy of Radix Saposhnikoviae is closely related to the content of secondary metabolites, so the optimal group of Radix Saposhnikoviae with the combined increase of the four chromones, was selected as the high-quality Radix Saposhnikoviae herb group, i.e., 0.5 mmol/L SNP treatment.

The ordinary Radix Saposhnikoviae group (0-day group) and the high-quality Radix Saposhnikoviae group (0.5 mmol/L SNP group) were dried to constant weight, taken 9.0 g, added 10 times the amount of water, soaked for 1 h, decocted for 1 h, filtered, and the extraction was repeated three times and the filtrates were combined. Concentrated to 0.045 g botanicals/mL, and 2.0 mL solution was given by gavage to each rat and concentrated 0.065 g botanicals/mL for mice at 0.2 mL each.

#### Animals

Animal experiments were conducted in accordance with the guidelines of the National Institutes of Health (NIH guidelines) and ARRIVE guidelines and approved by the Ethical Committee of Heilongjiang University of Chinese Medicine (approval number: HUCM2014-00348).

#### Antipyretic effect

In male SD rats, 180 ± 20 g, the basal body temperature was first measured 3 times a day for 3 days. For rats with an average anal temperature of 36.85 ± 0.37 °C, 2.0 mL/100 g of 15% yeast suspension were injected subcutaneously, and those whose body temperature increased by > 0.8 °C were selected as the test rats. The rats were randomly divided into four groups: saline group, model group, ordinary Radix Saposhnikoviae group, and high-quality Radix Saposhnikoviae group, with 10 rats in each group. The saline and model groups were administrated at 2.0 mL of saline daily, and the Radix Saposhnikoviae group was administrated at 0.045 g botanicals/mL for 2.0 mL daily for seven days. At the 3rd hour after the last administration, the rats were subcutaneously injected with 2.0 mL/100 g 15% yeast suspension, and their rectal temperature was measured at 0.5, 1, 2, 3, 4, and 5 h after injection.

#### Analgesic effect

Male Kunming mice, weighing 18 ± 2 g, were randomly divided into four groups: saline group, model group, ordinary Radix Saposhnikoviae group, and high-quality Radix Saposhnikoviae group, 10 mice in each group. 2.0 mL of saline was administrated daily for the saline group and the model group, and 0.2 mL 0.065 g botanicals/mL was administrated for the Radix Saposhnikoviae group. This operating process was done once a day for 7 consecutive days. At 3rd hour after the last administration, 0.2 mL 0.6% of acetic acid was injected intraperitoneally, and the number of twists in mice within 15 min after the injection was observed, and the twist inhibition rate was calculated.

Inhibition rate = [(numbers of twists in the control group − numbers of twists in assay group)/numbers of twists in control group] × 100%.

#### Determination of anti-inflammatory effect and inflammatory factors

Animal experiments were conducted in accordance with the guidelines of the National Institutes of Health (NIH guidelines) and approved by the Ethical Committee of Heilongjiang University of Chinese Medicine (approval number: HUCM2014-00348). Male Kunming mice weighing 18 ± 2 g were randomly divided into four groups: saline group, model group, ordinary Radix Saposhnikoviae group, and high-quality Radix Saposhnikoviae group, 10 mice for each group. 2.0 mL of saline was administrated daily for the saline group and the model group, and 0.2 mL was administrated for each Radix Saposhnikoviae group according to 0.65 g of botanicals/kg, once a day for 7 days. At the 3rd hour after the final administration, 0.03 mL of xylene was evenly applied to the anterior and posterior sides of the right ear of each mouse for 1 h to induce inflammation.

Both ears were cut along the baseline of the auricle, and the tissue of the same part of the left and right ear was weighed by punching with an 8 mm punch, and the swelling rate and swelling inhibition rate of the auricle of mice were calculated.

Swelling inhibition rate (%) = (mean swelling rate in the control group − mean swelling rate in the medicated group)/mean swelling rate in the control group × 100%

The contents of TNF-α and IL-6 were determined using the double antibody sandwich method with TNF-α and IL-6 kits.

### Data processing

Microsoft Office Excel 2007 and SPSS 26.0 software were used to process the data, and GraphPad Prism software was used to make graphs. The measurement data were expressed as ($\overline{x}\, \pm$  s) and analyzed by one-way variance analysis and t-test. *P* < 0.05 indicated statistically significant differences, while *P* < 0.01 indicated extremely statistically significant differences.

### Ethics declarations

Ethics approval to conduct animal experiments were conducted in accordance with the guidelines of the National Institutes of Health (NIH guidelines), ARRIVE guidelines and approved by the Ethical Committee of Heilongjiang University of Chinese Medicine (approval number: HUCM2014-00348).

## Results

### NO contents

**Figure 1:**
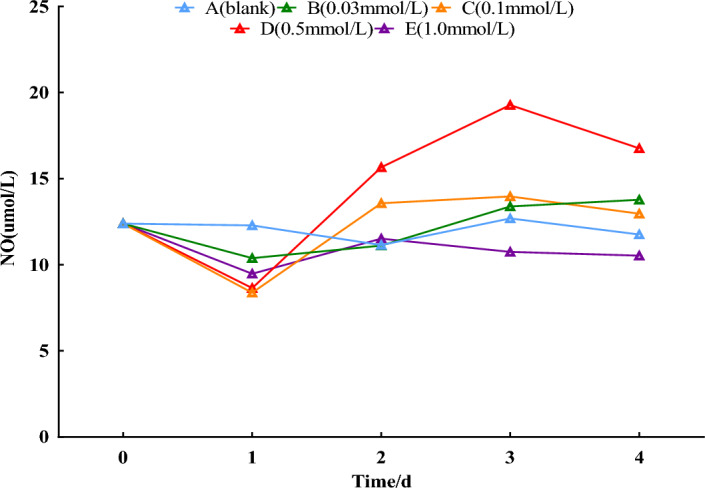
Effect of SNP on NO contents. The changes in NO contents were analyzed by spraying 0, 0.03, 0.1, 0.5, and 1.0 mmol/L SNP on the fresh roots of *S. divaricata* for two days and distilled water for the last three days.

Compared with the 0-day, there was no significant trend in the distilled water group and no significant trend in the 0.03, 0.1, and 1.0 mmol/L treatment groups except for a slight decrease on the 1st day. The 0.5 mmol/L SNP group caused a slight decrease in NO content in the fresh roots of *S. divaricata* on the first day and an increase on the 2nd and the 3rd day, reaching a peak on the 3rd day, with a rise of 55.0% compared with the 0-day (*P* < 0.01). As shown in Fig. 1.

### O_2_^·−^·contents

**Figure 2:**
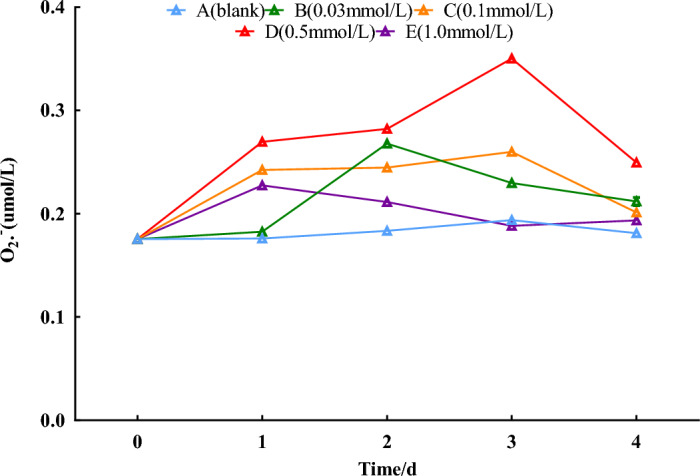
Effect of SNP on O_2_^**·**−^·contents. The changes in O_2_^**·**−^ contents were analyzed by spraying 0, 0.03, 0.1, 0.5, and 1.0 mmol/L SNP on the fresh roots of *S. divaricata* for 2 days and distilled water for the last 3 days.

Compared with the 0-day control, different concentrations of SNP all increased the O_2_^**·**−^ contents in the fresh roots of *S. divaricata*, showing a trend of increasing and then decreasing. The distilled water group showed no significant trend. The 0.03 and 1.0 mmol/L SNP groups peaked on the 2nd day. The 0.1 and 0.5 mmol/L treatment groups peaked on the 3rd day. The 0.5 group showed the most significant increase, with an increase of 135.3% compared with the 0-day (*P* < 0.01). As shown in Fig. 2.

### H_2_O_2_ contents

**Figure 3:**
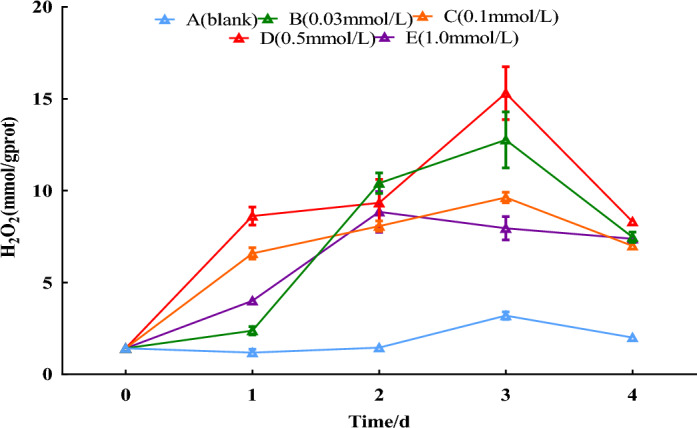
Effect of SNP on H_2_O_2_ contents. The changes in H_2_O_2_ contents were analyzed by spraying 0, 0.03, 0.1, 0.5, and 1.0 mmol/L SNP on the fresh roots of *S. divaricata* for 2 days and distilled water for the last 3 days.

Compared with the 0-day control, except for the distilled water group, the other groups showed no significant trend, the other H_2_O_2_ contents in the fresh roots of *S. divaricata* increased significantly, increasing and then decreasing from the 0 to the 4th day. The 1.0 mmol/L treatment group peaked on the 2nd day, and all other treatment groups peaked on the 3rd day. The 0.5 mmol/L treatment group showed a 985.1% increase (*P* < 0.01). As shown in Fig. 3.

### MDA contents

**Figure 4:**
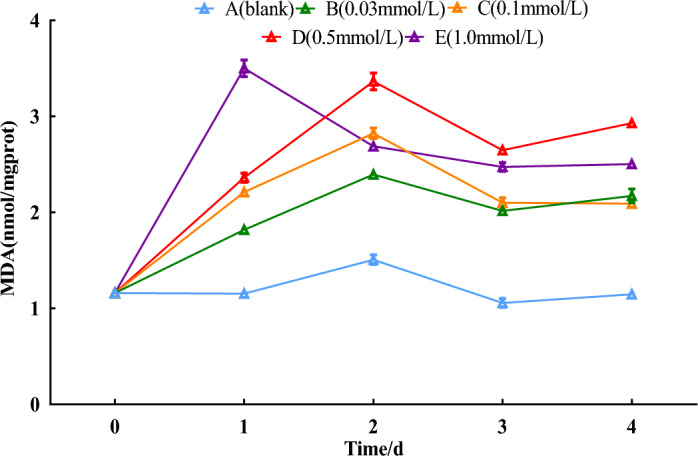
Effect of SNP on MDA contents. The changes in MDA contents were analyzed by spraying 0, 0.03, 0.1, 0.5, and 1.0 mmol/L SNP on the fresh roots of*S. divaricata* for 2 days and distilled water for the last 3 days.

Compared with the 0-day control, the MDA contents in the fresh roots of *S. divaricata* rose with concentrations of SNP, except for the distilled water group. The MDA contents increased as the concentration of SNP increased, with the 1.0 mmol/L treatment group reaching a peak on the 1st day and all other treatment groups reaching a peak on the 2nd day, with the most significant increase of 133.6% in the 0.5 mmol/L treatment group compared with the 0-day (*P* < 0.05). As shown in Fig. 4.

### Antioxidant enzyme activities

**Figure 5:**
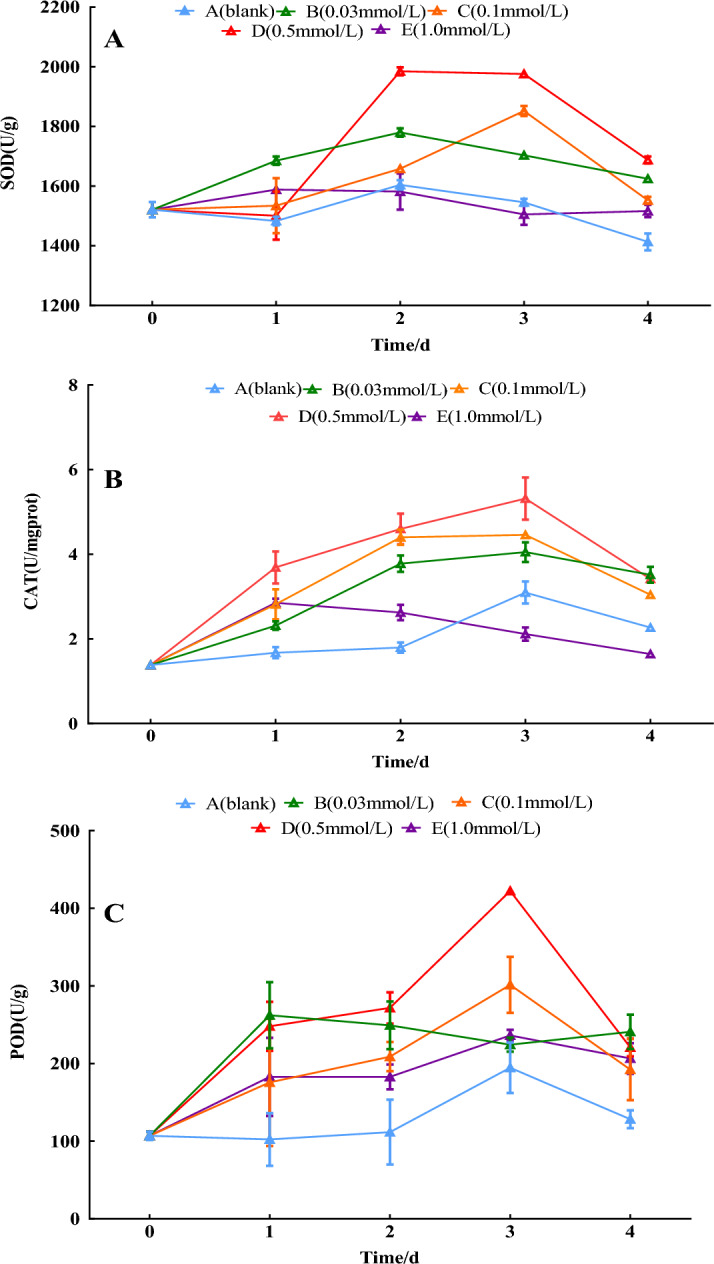
Effect of SNP on antioxidant enzyme activities. The changes in SOD, CAT and POD activities were analyzed by spraying 0, 0.03, 0.1, 0.5, and 1.0 mmol/L SNP on the fresh roots of *S. divaricata* for 2 days and distilled water for the last 3 days.

Compared with the 0-day control, the antioxidant enzyme activities of all treatment groups showed a trend of increasing and then decreasing, except for the distilled water group and the 1.0 mmol/L treatment group, with a minor variation in antioxidant enzyme activities. SOD peaked on the 2nd day, with a 27.5% increase in the 0.5 mmol/L treatment group. CAT and POD activity peaked on the 3rd day, with 281.6% and 297.1% increases in the 0.5 mmol/L group, respectively (*P* < 0.05). As shown in Fig. 5.

### Metabolic pathway

#### 1,3-DPG contents

**Figure 6:**
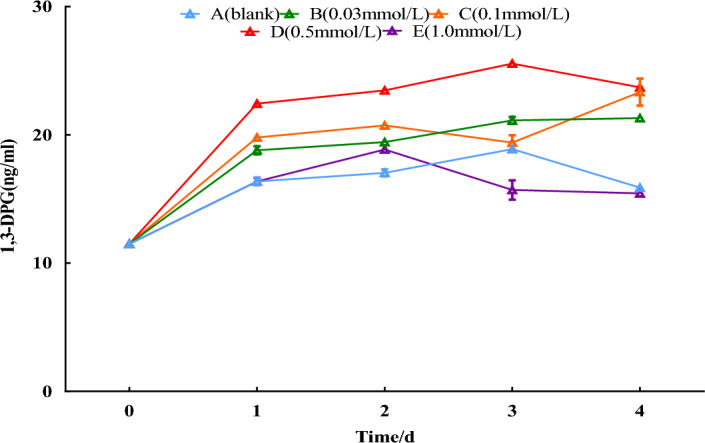
Effect of SNP on the contents of primary metabolites. The changes in 1,3DPG contents were analyzed by spraying 0, 0.03, 0.1, 0.5, and 1.0 mmol/L SNP on the fresh roots of *S. divaricata* for 2 days and distilled water for the last 3 days.

Compared with 0-day, there was no significant trend in the distilled water group and 1.0 mmol/L group, while the other SNP group showed an upward trend. Among them, the 0.03 and 0.5 mmol/L groups peaked on the 3rd day, increasing by 83.5% and 121.7%, respectively, compared with the 0-day. As shown in Fig. 6.

#### Key enzyme activities related to secondary metabolites

**Figure 7:**
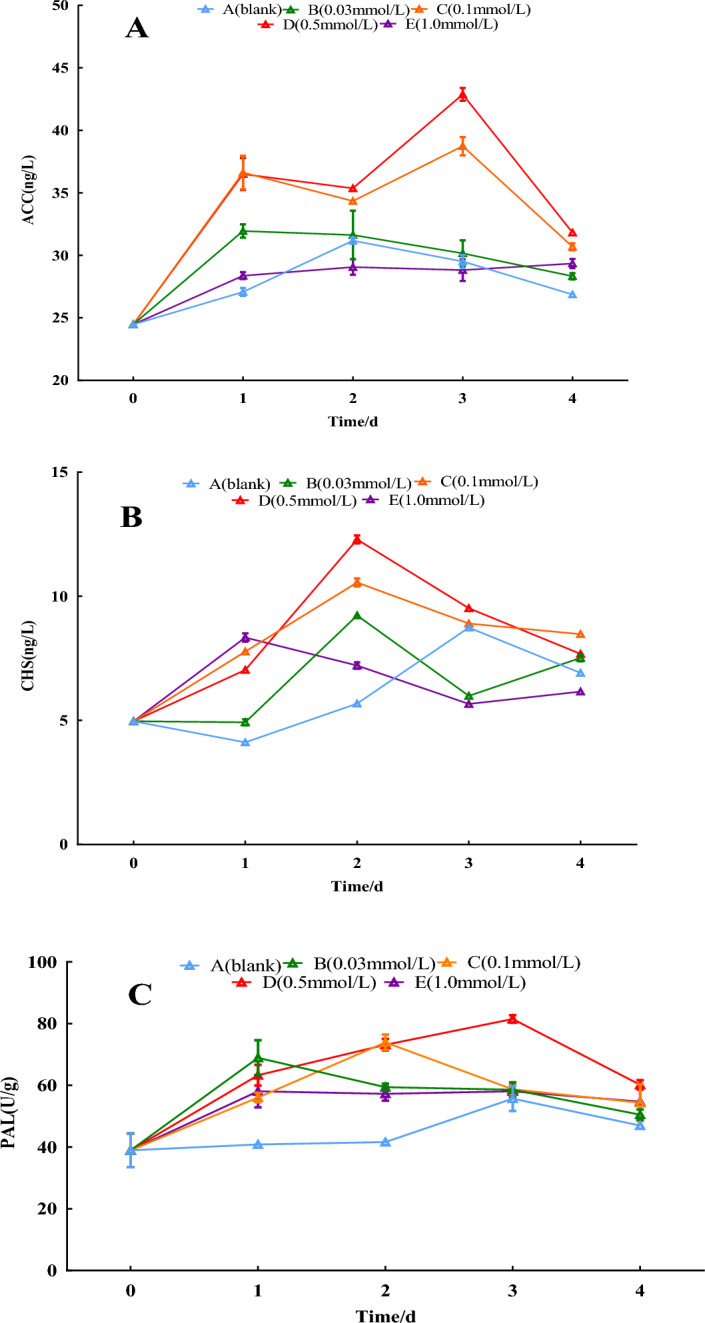
Effects of SNP on key enzyme activities relating to secondary metabolites. The changes in ACC, CHS and PAL activities were analyzed by spraying 0, 0.03, 0.1, 0.5, and 1.0 mmol/L SNP on the fresh roots of *S. divaricata* for 2 days and distilled water for the last 3 days.

The PAL increased slightly on the 3rd day in the distilled water group compared with the 0-day. Except for the 1.0 mmol/L treatment group, all treatment groups peaked successively later with increasing concentrations of SNP, 0.03, 0.1, and 0.5 mmol/L SNP treatment groups peaking on the 1st, 2nd, and 3rd day, respectively. The 0.5 mmol/L treatment group showed a 119.7% increase on the 3rd day compared with the 0-day. CHS increased in all groups compared with the 0-day. All groups reached a peak on the 2nd day except the distilled water, and the 1.0 mmol/L SNP group peaked on the 2nd day. The 0.5 mmol/L SNP-treated group showed a 114.0% increase compared with the 0-day. Compared with the 0-day, ACC has slightly elevated but a minor variation change in the distilled water group, 0.03, and 1.0 mmol/L SNP-treated groups. The 0.5 mmol/L SNP-treated groups showed a 74.7% increase compared with the 0-day. As shown in Fig. 7.

#### Secondary metabolites contents

**Figure 8:**
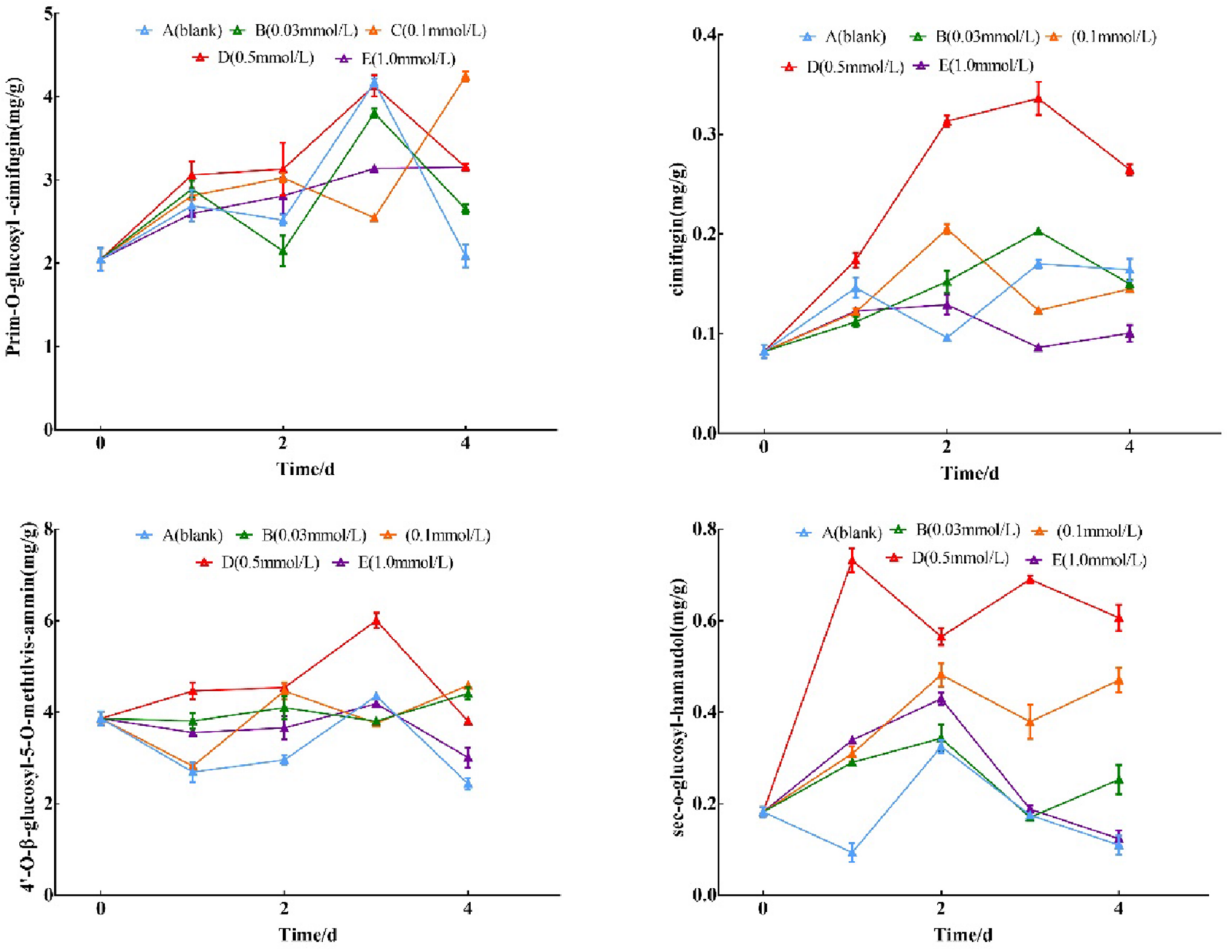
Effect of SNP on the contents of secondary metabolite. The changes in secondary metabolite contents were analyzed by spraying 0, 0.03, 0.1, 0.5, and 1.0 mmol/L SNP on the fresh roots of *S. divaricata* for 2 days and distilled water for the last 3 days.

The contents of prim-*O*-glucosylcimifugin, cimifugin, 4′-*O*-β-d-glucosyl-5-*O*-methylvisamminol, and sec-*O*-glucosylhamaudol were increased by SNP in the Radix Saposhnikoviae, with a trend of increasing and then decreasing in each treatment group, among which the total content of four chromones had the highest increase for the 0.5 mmol/L on the 3rd day, prim-*O*-glucosylcimifugin with a rise of 88.3%, 4′-*O*-β-d-glucosyl-5-*O*-methylvisamminol with 55.4%, cimifugin with 325.0% and sec-*O*-glucosyl hamaudol with 283.8%, compared with the control group, respectively. As shown in Fig. 8.

### Pharmacological effects verification

#### Antipyretic effect

Compared with the model group, the body temperature of rats in Radix Saposhnikoviae groups was reduced after the 2nd day. Still, the high-quality Radix Saposhnikoviae group was lower significantly than the ordinary Radix Saposhnikoviae group. As shown in Table 1.

**Table 1:** Comparison of body temperature of dry yeast-induced fever rats in each group ($\overline{x}\, \pm \,$ s).

Group	Basal body temperature	After administration					
		0.5 h	1 h	2 h	3 h	4 h	5 h
1	37.16 ± 0.24	37.36 ± 0.33	37.36 ± 0.36	37.43 ± 0.33	37.45 ± 0.34	37.46 ± 0.16	37.28 ± 0.38
2	37.01 ± 0.39*	37.4 ± 0.4**	37.55 ± 0.35*	37.57 ± 0.23**	37.94 ± 0.84	38.45 ± 0.35	38.64 ± 0.54
3	36.98 ± 0.62*^,##^	37.5 ± 0.6**^,#^	37.55 ± 0.25*	37.21 ± 0.61*	37.78 ± 0.42^#^	38.05 ± 0.25*	37.68 ± 0.32
4	36.99 ± 0.38*^,##^	36.78 ± 0.52	36.72 ± 0.48	36.9 ± 0.3	37.37 ± 0.23**	37.94 ± 0.36^#^	37.55 ± 0.45*^,#^

Compared with the blank group, *P < 0.05, **P < 0.01; compared with the model group, ^#^P < 0.05, ^##^P < 0.01.

(1) Blank group; (2) Model group; (3) Ordinary Radix saposhnikoviae*.*; (4) High-quality Radix saposhnikoviae.

The groups were administered by gavage for 7 days, and 3 h after the last administration, the changes in body temperature of rats at 0.5, 1, 2, 3, 4, and 5 h after the injection of dry yeast were greatly observed and analyzed.

#### Analgesic effects

Compared with the model group, the number of twisting in mice in the Radix Saposhnikoviae group was significantly reduced. Still, the reduction in the high-quality Radix Saposhnikoviae group was lower than that in the ordinary Radix Saposhnikoviae group; the number of twisting was decreased by 4.17 times on average, and the inhibition rate was increased by 14.45%, which was 1.5 times of the ordinary botanicals. As shown in Table 2.

**Table 2:** Comparison of twisting times in mice of each group ($\overline{x}\, \pm \,$ s).

Group	Twisting times (times)	Inhibition rate (%)
1	–	–
2	28.85 ± 7.15	–
3	18.50 ± 5.50^##^	35.88%
4	14.33 ± 4.67^##^	50.33%

Compared with the blank group, *P < 0.05, **P < 0.01; compared with the model group, ^#^P < 0.05, ^##^P < 0.01.

(1) Blank group; (2) Model group; (3.) Ordinary Radix saposhnikoviae; (4) High-quality Radix saposhnikoviae.

Each group was administered by gavage for 7 days, and 3 h after the last administration, the number of writhing times in mice injected with acetic acid within 15 min was observed and analyzed.

#### Anti-inflammatory effect and inflammatory factors

Compared with the model group, the ear swelling of mice in the Radix Saposhnikoviae group was significantly reduced; among them, the reduction was more significant in the high-quality Radix Saposhnikoviae group than in the ordinary Radix Saposhnikoviae group, with a reduction of 1.18 mg in weight and an increase of 38.78% in the inhibition rate which was 3.1 times higher than that of the ordinary botanicals. The two inflammatory factors, IF-6 and TNF-α were significantly reduced in the Radix Saposhnikoviae group compared with the model group. Still, the high-quality Radix Saposhnikoviae group had a significantly higher degree of reduction than the ordinary Radix Saposhnikoviae group, with a decrease of 17.52% and 18.00%, respectively, and the contents of inflammatory factors in the high-quality Radix Saposhnikoviae group were consistent with that in the blank group, indicating excellent therapeutic efficacy of the high-quality group. As shown in Table 3.

**Table 3:** Comparison of anti-inflammatory effects among different groups of mice.

Group	Ear swelling degree		Inflammatory factors	
	Swelling degree (mg)	Inhibition rate (%)	IL-6 content (pg/mL)	TNF-α content (ng/L)
1	–	–	17.07 ± 2.86	128.0 ± 7.5
2	4.27 ± 1.07	–	22.20 ± 1.02*	167.8 ± 15.0*
3	2.66 ± 0.66^#^	18.75%	20.23 ± 1.63*^,##^	155.0 ± 13.2*^,#^
4	1.48 ± 1.11^#^	57.52%	16.34 ± 3.23*^,#^	124.8 ± 7.3*^,##^

Compared with the blank group, *P < 0.05, **P < 0.01; compared with the model group, ^#^P < 0.05, ^##^P < 0.01.

(1) Blank group; (2) Model group; (3) Ordinary Radix saposhnikoviae; (4) High-quality Radix saposhnikoviae.

Each group was administered the drug by gavage for 7 days, and 3 h after the last administration, the swelling degree of mice's ears was observed after xylene was applied for 1 h, and mice's blood was collected and analyzed for the content of IL-6 and TNF-α.

## Discussion

### Effects of SNP on NO, ROS, and MDA

SNP was used as an exogenous donor of NO, which resulted in a significant increase of NO contents; the 0.5 mmol/L group with a marked difference reached a peak on the 3rd day (Fig. 1). Under the effect of NO, O_2_^**·**−^ contents also continued to increase from the 1st to the 3rd day (Fig. 2), and the excess O_2_^**·**−^ was converted into H_2_O_2_ by SOD, resulting in a rise in H_2_O_2_ contents during the same period from the 1st to the 3rd day (Fig. 3). MDA as product of the destroyed bio-membrane can directly reflect ROS damage to plant cells^28^. In this study, the MDA contents remained consistently high from the 2nd day (Fig. 4). On the 4th day, the levels of NO, O_2_^**·**−^, and H_2_O_2_ were all decreased, probably due to NO's generation of more O_2_^**·**−^, which might have caused damage to corresponding enzymes. O_2_^**·**−^, H_2_O_2,_ and MDA as indexes of ecological stress were significantly increased (Fig. 4), indicating that SNP can induce a physiological response of *S. divaricata* under ecological stress*.*

### Effects of SNP on antioxidant enzyme activities

When the ROS content is too high, it can damage nearby molecular structures, lipid bilayer, DNA single strands, proteins, etc. The strategy of living organisms avoiding ROS damage is to evolve antioxidant substances to eliminate excess ROS. Antioxidant enzymes are a large group of substances that eliminate ROS in organisms. The biosynthesis and activities of these enzymes are induced and enhanced by the presence of ROS^29^. As the most significant nonspecific antioxidant substances, these enzymes play a crucial role in maintaining cellular redox balance and protecting cells from oxidative damage caused by ROS. SOD, as the primary defense enzyme, can convert O_2_^**·**−^ into H_2_O_2,_ then, other antioxidant enzymes, such as catalase (CAT) and peroxidase (POD), work together to convert H_2_O_2_ into H_2_O and O_2_^30^. SOD peaked on the 2nd day, converting O_2_^**·**−^ into H_2_O_2_, and H_2_O_2_ contents were significantly increased in the 0.1 and 0.5 mmol/L SNP-treated groups on the 3rd day. Meanwhile, CAT and POD activities also elevated, especially in the 0.5 mmol/L group (*P ˂ *0.05). With the interaction of CAT and POD, H_2_O_2_ contents decreased on the 4th day (Fig. 5), O_2_^**·**−^ and H_2_O_2_ contents and antioxidant enzyme activities decreased, probably resulting from increased ROS level (Figs. 2, 3, 5), which caused damage to cellular proteins^31^.

### Effect of SNP on secondary metabolite and relating key enzymes

Antioxidant enzymes are proteins with –S–S-bonds and other chemical bonds that maintain the corresponding structure, allowing them to function as catalysts in neutralizing ROS. However, these chemical bonds are unstable, excessive ROS can easily alter the configuration of antioxidant enzymes and affect activities^32^. Plants face more ecological stresses and generate more ROS due to their immobility. Studies have demonstrated that under severe ecological stress, even in highly adaptive plants like *Glycyrrhiza uralensis* Fisch, the activities of antioxidant enzymes such as SOD, CAT, and POD are also drastically reduced^33^, not to mention other plants. Thus, relying solely on antioxidant enzymes is impossible for plants to adapt to severe ecological stress. Adaptive strategies for plants are that they evolved the secondary metabolism as a supplement.

**Figure 9:**
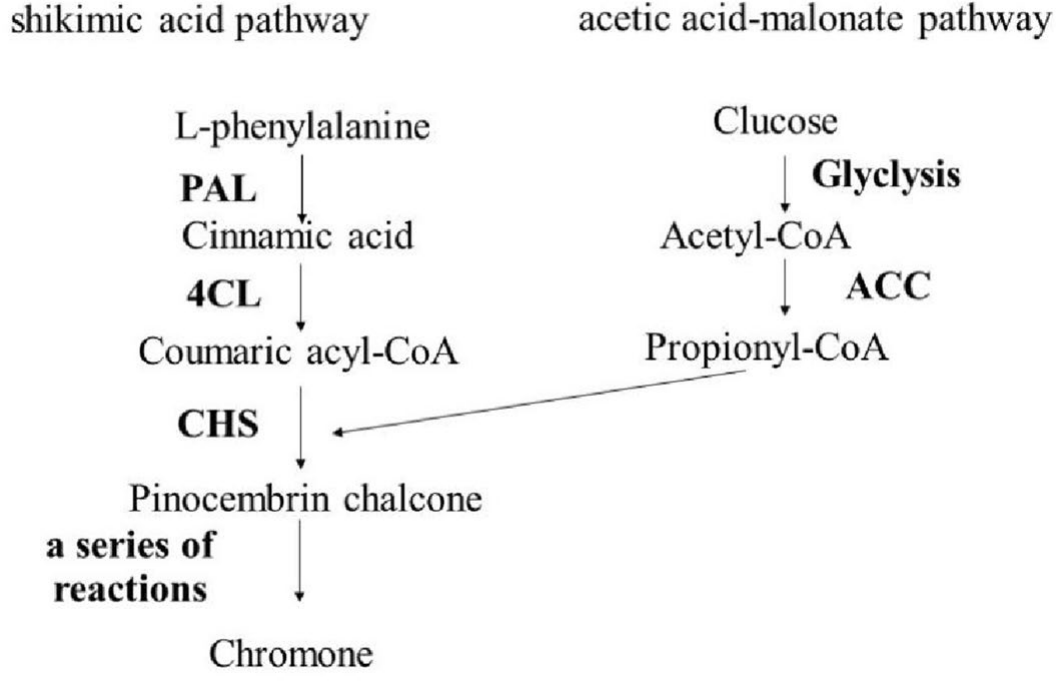
Synthesis path diagram of Radix Saposhnikoviae chromones. Analysis of the synthesis of secondary metabolites of Radix Saposhnikoviae via shikimic acid pathway and acetic acid-malonate acid pathway and the key enzymes involved in the synthesis.

The main secondary metabolites of *S. divaricata* are chromones, which have two biosynthetic pathways. One is the acetic acid-malonate pathway, in which the acetyl-CoA carboxylation enzyme (ACC) is a key and limiting enzyme that catalyzes the carboxylation of acetyl-CoA to form propionyl-CoA. The other is the shikimic acid pathway, in which phenylalanine ammonia-lyase (PAL), a key enzyme for flavonoids, catalyzes phenylalanine to form cinnamic acid. Coumaric acid under cinnamate 4-hydroxylase (C4H), followed by coumaric acyl-CoA under 4-coumaroyl-CoA ligase (4CL). Based on this, the propionyl-CoA and the coumaric acyl-CoA undergo a series of reactions under chalcone synthase and form chromone compounds^34–36^. As shown in Fig. 9.

The activities of the key enzymes relating to secondary metabolism could be significantly increased by increasing ROS and NO itself^37^. By applying SNP to the fresh roots, the activities of ACC, PAL, and CHS, the key enzymes of secondary metabolite biosynthesis, were increased in each treatment group, among them, the activities of CHS and ACC in the 0.5 mmol/L SNP group increased by 117.8% and 44.1% on the 2nd day and 3rd day, respectively (Fig. 7), resulting in enhancement of chromone compounds biosynthesis. For 0.5 mmol/L SNP-treated on the 3rd day, the contents of prim-*O*-glucosylcimifugin rose from 2.05 to 4.13 mg/g, cimifugin from 0.08 to 0.34 mg/g, 4′-*O*-β-d-glucosyl-5-*O*-methylvisamminol from 3.86 to 6.00 mg/g, and sec-O-glucosylhamaudol from 0.18 to 4.13 mg/g, respectively, with a remarkable increase of 88.3%, 325.0%, 55.4%, and 283.8%, respectively. 1,3-DPG is a product of glucose catabolism in glycolysis and a material of glucose anabolism in gluconeogenesis. This study must derive from glucose since the separated fresh roots are being used. The exogenous SNP promoted glucose catabolism, provided raw materials for the biosynthesis of secondary metabolites, and ensured that the various chromone contents increased from the 1st to the 3rd day. Under mild ecological stress, the ROS contents were relatively low, and the antioxidant enzymes can exert a more significant antioxidant effect; however, under severe ecological stress, solely antioxidant enzymes have difficulty coping with too much ROS due to reduced activities by ROS, the secondary metabolites would play an important role^38^. The lower concentrations of 0.03 and 0.1 mmol/L SNP groups induced less ROS (Figs. 2, 3) and lower levels of antioxidant enzymes, key enzyme activities of secondary metabolism, which resulted in secondary metabolites being lower (Figs. 5, 7, 8). In the 1.0 mmol/L SNP-treated group, the highest level of MDA had been hit on the 2nd day. It failed to produce high levels of ROS, possibly due to the damaging effect of high levels of NO^39^ or/and reduced the enzymes related to ROS production or/and produced a high amount of ROS^36^, which resulted in the secondary metabolites failing to be elevated.

1,3-DPG is a product of glucose catabolism. ACC and PAL serve as intermediaries between primary and secondary metabolism, while CHS is a critical enzyme in plant flavonoid biosynthesis. The presence of a series of substances indicated that the increased chromones come from biosynthesis, not biotransformation. This ensures that the various active ingredients are significantly increased.

### Validation of pharmacodynamics

Botanicals contain a wide variety of active ingredients, and there are significant variations in the contents, activities, and bio-availabilities of various active ingredients^40^. Due to such diversity, it is difficult to objectively evaluate the quality of botanicals only by the contents of a few ingredients or certain ingredients^41^. Pharmacodynamics is the best method to obtain a comprehensive and accurate assessment of botanical quality. The main effects of Radix Saposhnikoviae are antipyretic, analgesic, and anti-inflammatory^2^, and the main active ingredients are chromones^42^. For 0.5 mmol/L SNP on the 3rd day, the contents of prim-*O*-glucosylcimifugin, cimifugin, 4′-*O*-β-d-glucosyl-5-*O*-methylvisamminol, and sec-*O* -glucosylhamaudol increased by 88.3%, 325.0%, 55.4%, and 283.8%, respectively, especially, the improved high-active components such as cimifugin and sec-*O*-glucosylhamaudol were even more remarkable. Cimifugin is easier to enter the cell membrane to play a drug effect due to more –OH and is stronger lipophilic than glycosides^41,43^; the pharmacological effects of sec-*O*-glucosylhamaudol are also stronger due to more –OH than prim-*O*-glucosylcimifugin and 4′-*O*-β-d-glucosyl-5-*O*-methylvisamminol^44^. With this, compared with the model group, the body temperature of the high-quality Radix Saposhnikoviae group (0.5 mmol/L SNP group) was reduced by 0.11–0.13 °C. The inhibition rate of twisting body and ear swelling in mice was increased by 14.45% and 38.78%, the inhibition rate being 1.5 and 3.1 times of the ordinary botanicals, respectively. The contents of IF-6 and TNF-α were reduced by 17.52% and 18.00%, and the inflammatory factor content fell into the original level of the blank group, which indicated that the efficacy of the high-quality Radix Saposhnikoviae group was significantly improved.

The nano-materials as delivery carriers have been used for biological research for decades, and some significant breakthroughs have been made^45^. For example, Cerium oxide nanoparticles increase NO production in rice leaves under salt stress and enhance nitrate reductase activity and nitrate reductase activity^46^. Similarly, multilayer nanotubes have shown the ability to modulate NO production and improve salt tolerance in oilseed rape^47^. SNP is an injectable agent for the treatment of hypertension and acute heart failure, the application to herb quality improvement is also in very low concentrations and amounts. Besides, the SNP only increases the level of intrinsic ingredients of *S. divaricata* under drought stress without producing other toxic components. Therefore, it is safe to use SNP. SNP as an inducer of secondary metabolism has promising applications.

## Conclusion

The exogenous NO donor SNP can increase the levels of ROS, resulting in the enhancement of antioxidant enzyme activities and secondary metabolite contents in fresh roots. The antioxidant enzymes and secondary metabolites work together and reduce the damage to the plant body caused by ROS. In the 0.5 mmol/L SNP-treated group, prim-O-glucosylcimifugin, cimifugin, 4′-*O*-β-d-glucosyl-5-*O*-methyl visamminol, and sec-*O*-glucosylhamaudol increased by 88.3%, 325.0%, 55.4%, and 283.8%, respectively. Based on this, the efficacy of Radix saposhnikoviae was enhanced. This study is based on a common strategy for plants to adapt to ecological stresses, applying SNP to improve the quality of Radix Saposhnikoviae can provide a new pathway for producing other high-quality medicinal herbs.

### Supplementary Information

Supplementary Information 1.

Supplementary Information 2.

## Data Availability

The data that support the findings of this study are available from the corresponding author (TI)upon request. Source data are provided with this paper.
