# Supplementary material for: Sodium nitroprusside improved the quality of Radix Saposhnikoviae through constructed physiological response under ecological stress
Source: Sci Rep. 2023 Sep 22;13:15823. doi: 10.1038/s41598-023-43153-3 (PMC10516912; doi:10.1038/s41598-023-43153-3)
Supplement: Supplementary file 1 — Supplementary Information 1. [file 41598_2023_43153_MOESM1_ESM.docx]

**
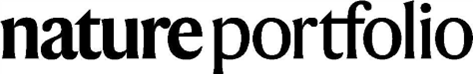
**

**
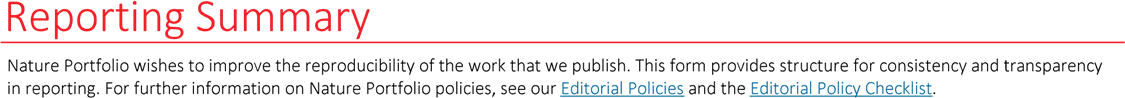
**

**Animals and other research organisms**

Policy information about studies involving animals; ARRIVE guidelines recommended for reporting animal research, and Sex and Gender in

Research

Laboratory animals

Laboratory animals were obtained from the Center for Drug Safety Evaluation of Heilongjiang University of Chinese Medicine. The experimental animals use feed and litter from Liaoning Changsheng Biotechnology Co., Ltd. feed and bedding is appropriately packaged to ensure the integrity of the contents during storage.Mouse cages include a protective filter that prevent the introduction of dust, dander, dirt and potential disease-causing organisms into the cage. Cages and other equipment are cleaned and replaced as often as needed to maintain clean, dry housing for the animals. Animals are housed on Individual ventilated racks. SD rat & Kunming mouse are housed 10 animals per cage, cages with different models.Mice are fed with SD rat & Kunming mouse pellets and water is provided. Feed and water are dispensed in to sterile containers in a laminar flow cabinet, and water is provided ad libitum. Animal rooms are maintained at ventilation (min 16 air changes per hour), temperature between 18℃ and 24℃ and relative humidity between 50% and 70%. All rooms are operated with automatic timers to maintain 12 hours: 12 hours light/dark cycle.

Wild animals

No wild animals were used in the study.

Reporting on sex

Although we don't have sex bias, we have used male SD rats & Kunming mice in the study because female mice tend to instability. Due to instability nature of the female mice, they have been excluded from the studies.

Field-collected samples

No field collected samples were used in the study.

Ethics oversight

Ethics approval to conduct animal experiments were conducted in accordance with the guidelines of the National Institutes of Health (NIH guidelines), ARRIVE guidelines and approved by the Ethical Committee of Heilongjiang University of Chinese Medicine (approval number: HUCM2014-00348).

Note that full information on the approval of the study protocol must also be provided in the manuscript.
